# Emerging health risks from agricultural intensification in Southeast Asia: a systematic review

**DOI:** 10.1080/10773525.2018.1450923

**Published:** 2018-03-21

**Authors:** Steven Lam, Giang Pham, Hung Nguyen-Viet

**Affiliations:** a Hanoi University of Public Health, Center for Public Health and Ecosystem Research, Hanoi, Vietnam; b Department of Population Medicine, University of Guelph, Guelph, ON, Canada; c Vietnam Public Health Association, Hanoi, Vietnam; d International Livestock Research Institute, Hanoi, Vietnam

**Keywords:** Agricultural intensification, crop production, health risk, livestock production, Southeast Asia, systematic review

## Abstract

**Background:**

Agricultural intensification is having profound impacts on food security and rural livelihoods; however, concerns remain about the potential implications on public health.

**Objectives:**

We aim to examine and synthesize the evidence for human health risks of agricultural intensification in Southeast Asia.

**Methods:**

We conducted a systematic review of peer-reviewed articles published between January 2000 and December 2015 from two electronic databases (PubMed, CAB Direct).

**Results:**

A total of 73 relevant studies were included and evaluated. More than half of the studies used epidemiological methods while others applied alternative methods to quantify or estimate risks. Studies mainly focused on occupational and consumer exposure to pesticides, without often specifying the actual health risk.

**Conclusion:**

Overall, the current knowledge on health risks appears to be limited. More research on long-term health implications and a wider range of contaminants are needed if sustainable benefits are to be obtained from agricultural intensification.

## Introduction

With the world population predicted to reach nine billion by 2050, sustainably increasing food production systems to achieve global food security is a real challenge [1]. This is especially true for developing countries, where nearly all of the population growth is expected to occur [2]. Agriculture is a major source of livelihood in developing countries, especially for the rural poor, and nearly 75% of poor people live in rural areas [3]. Growth in agricultural production can support the livelihoods of many poor rural farmers [4], increase global food security, and help countries meet the sustainable development goals of ending poverty and hunger by 2030 [5].

Agricultural intensification, defined as the increase in the productivity of crops and livestock per unit of input [6], has grown rapidly in Southeast Asia driven by large population growth, strong economies, and a shift in consumer demand. Agriculture is the primary economic activity in Southeast Asia, and the share of agriculture to GDP, along with net agricultural output per capita, has more than doubled in the last few decades [7]. While agricultural intensification can support food security and socioeconomic development, the public health impacts are not yet well understood [8,9]. For instance, the intensive use of chemical fertilizers and pesticides for crop production can increase occupational exposure of farmers to chemical and pesticide residues, while also placing pressures on ecosystems through excess residues and toxins in the groundwater and surface water [10–12]. Furthermore, increased livestock production generates large amounts of waste and waste-by-products. Combined with outdated waste management technologies, there are potential health risks to farmers through occupational waste management practices, along with consumers through consumption of waste-contaminated products [9]. Intensive livestock production also accelerates greenhouse gas production and exacerbates climate change [13,14]. As such, the potential impacts of intensive agriculture on health are a growing concern.

Agriculture and health are intrinsically linked; but to what extent intensive agricultural practices can be made more sustainable and expanded, while protecting public health, is a global challenge. Going further, a better understanding of the conservation of ecosystem services in agricultural food systems is needed for sustainable agriculture, ecosystems, and protection of human health. In an effort to better understand the health risks associated with agricultural intensification in Southeast Asia, a systematic review was conducted.

## Methods

Methods followed standard guidelines for scoping and systematic reviews [15,16]. The core review team consisted of individuals with topic (agriculture, food safety, environmental health, public health) and methodological (knowledge synthesis) expertise. The systematic review included the following five key phases: (1) identifying the research question; (2) identifying relevant studies; (3) study selection; (4) charting the data; and (5) synthesizing and reporting the results.

### Research question

This review was guided by the question “What are the human health risks of agriculture intensification in Southeast Asia (Indonesia, Malaysia, Singapore, Brunei, Philippines, Vietnam, Cambodia, Timor Leste, Laos, Thailand, and Myanmar)?” The acronym PICO was used to frame the research question according to Population (e.g. people in Southeast Asia), Intervention (e.g. intensive crop or livestock production), Comparison (e.g. no intensive crop or livestock production), and Outcome (e.g. health). Since one review by Lam et al. was previously conducted on health risks of agricultural waste management in Southeast Asia from 2000 to 2014 [9], we did not replicate the literature search, except for the recent years (2014–2015).

### Data sources and search strategy

The search was conducted in April 2016 in two electronic databases: PubMed (http://www.ncbi.nlm.nih.gov/pubmed/) and CAB Direct (http://cabdirect.org). These databases were selected to be comprehensive and cover disciplines in health, agriculture, and environment. Limits on database search included peer-reviewed literature and English language. For contemporary reasons, we selected articles from 1 January 2000 to 31 December 2015. The search strategy employed broad search terms (Table 1) to ensure publications were not overlooked, and many publications were then excluded. All citations were imported into the web-based application DistillerSR (Evidence Partners Incorporated, Ottawa, ON) and duplicate citations were removed using the DistillerSR duplicate removal function. Relevance screenings and data characterization of full articles were subsequently performed using DistillerSR.

**Table 1.** Systematic review search strategy with algorithms for each database to identify peer-reviewed articles examining the human health risks of agriculture intensification in Southeast Asia.

Databases	Main terms	Expanded terms
PubMed, CAB Direct	Health	(“adverse effect” OR health OR disease OR death OR morbidity OR mortality OR pathogen OR illness OR ailment OR allerg OR allergies OR infection OR diarrhea OR “well-being” OR “well being”) **AND**
	Agriculture intensification	(“agricultural intensification” OR “intensification of agriculture” OR “crop intensification” or “intensive food production” OR “livestock intensification” OR “intensive farming” OR
	Waste management^a^	“agricultural waste” OR wastewater OR “waste water” OR “integrated waste” OR “faecal sludge” OR manure OR “animal waste” OR “solid waste” OR “human waste” OR “livestock waste” OR feces OR feces OR “animal waste” OR excreta OR excrement OR
	Agricultural inputs^b^	fertilizer OR irrigation OR “agricultural chemical” OR agrochemical OR (hormone AND agriculture) OR (antimicrobial AND agriculture) OR (antibiotic AND agriculture) OR (pesticide AND agriculture)) **AND**
	Location	(Brunei OR Cambodia OR Indonesia OR Laos OR Malaysia OR Myanmar OR Philippines OR Singapore OR Thailand OR “Timor Leste” OR “Viet Nam” OR Vietnam OR “Southeast Asia” OR “South East Asia”)

a Literature on waste management was limited to 2014 and 2015 as relevant studies from 2000–2014 were captured in a review by Lam et al. [9].

b Individual search terms for agricultural inputs (hormone, antimicrobial, antibiotics, pesticides) yielded many irrelevant studies. As such, these terms were combined with “agriculture.”

### Relevance screening and eligibility criteria

A two-step relevance screening strategy was employed. For the first level of screening, titles and abstracts of articles were screened for relevance; next, all citations deemed relevant went through a review of the full-text articles. Studies were eligible for inclusion if they explored agriculture intensification and human health outcomes; environmental or food contaminants with reference to a standard; or health risk assessments (Table 2). The title and abstract, as well as full text of each citation were independently screened by two reviewers. Reviewers met throughout the screening process to resolve conflicts and discuss any uncertainties related to study selection [17]. The inter-rater reliability, or degree of agreement among reviewers, was calculated [18].

**Table 2.** Inclusion and exclusion eligibility criteria applied during screening of articles to identify articles examining the human and ecosystem health risks of agricultural intensification in Southeast Asia.

Category	Inclusion	Exclusion
Geographic area	Southeast Asia	Any other region
Research topic	Intensive crop or livestock production with relevance to health outcome	Described crop or livestock production without relevance to health (and vice versa)
Publication date	1 January 2000 to 31 December 2015	Studies published before 2000
Study design	Original peer-reviewed articles published in English, including: epidemiological studies; studies of environmental or food contaminants with reference to a standard; and health risk assessments	Reviews, commentaries, theses

### Data charting

A form was developed by the authors to extract study characteristics (available upon request by the first author). The characteristics of each relevant full-text article were extracted by one reviewer. The data collection categories included: author, year of publication, geographic location, study design, exposure pathway, and health risks. The data were compiled in a single spreadsheet using DistillerSR report function and subsequently imported into Microsoft Excel 2010 (Microsoft Corporation, Redmond, WA) for synthesis.

### Summarizing and reporting

A narrative synthesis approach was used to provide an overview of the existing literature. Firstly, an overall summary of study findings was synthesized taking into account study variations that may affect generalizability of research results, such as variations in populations, study area, study design, and agricultural practice. Then, the study results were organized into categories using thematic analysis techniques [19].

## Results

### Overview of studies identified

**Figure 1.**
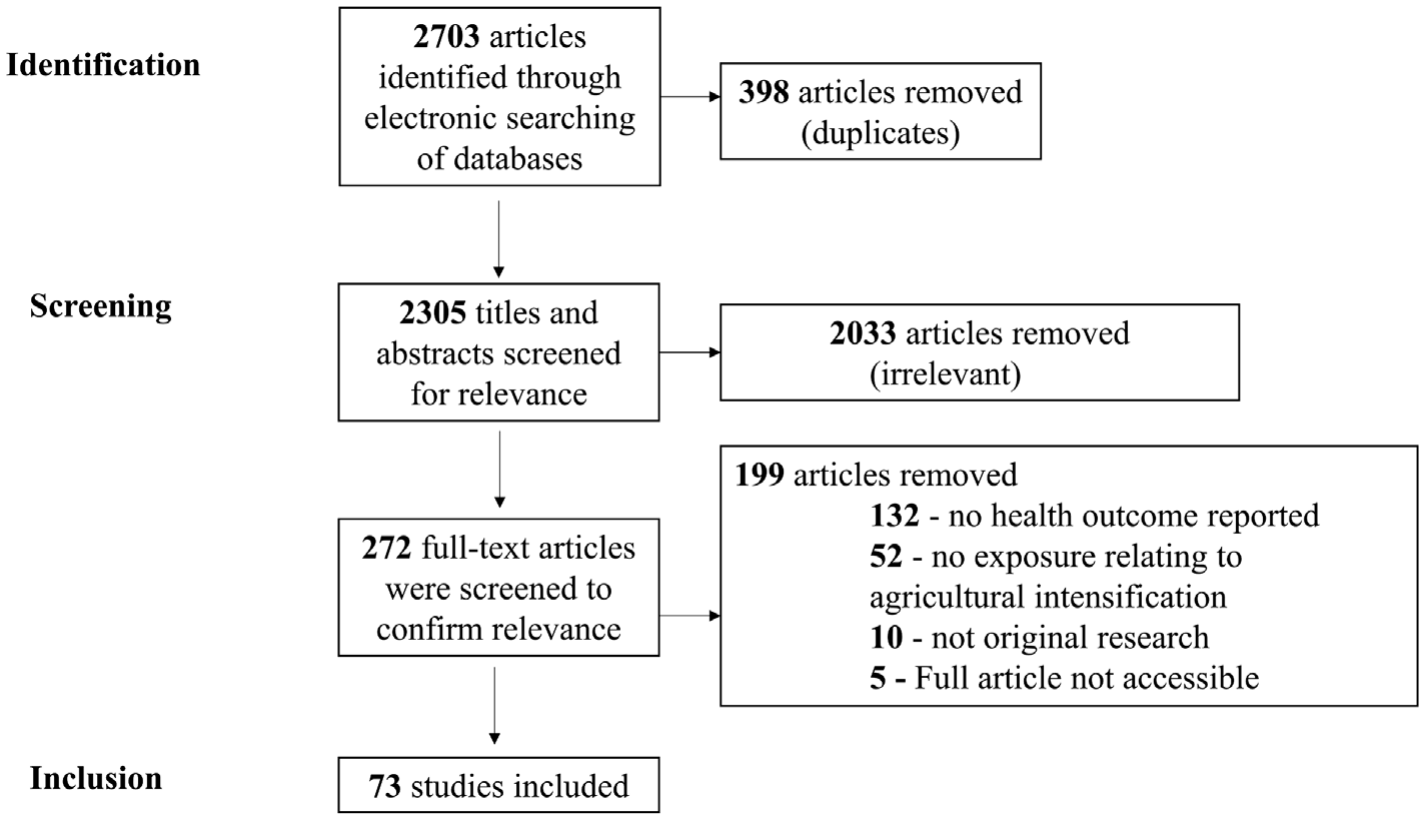
Flow chart of the selection of studies that examined the health risks of agricultural intensification in South-East Asia.

The search strategy identified 1271 studies in PubMed and 1432 studies in CAB Direct, totaling 2703 articles. Duplications were removed, resulting in 2305 unique citations. After primary title and abstract screening, 272 were included as potentially relevant. After examination of the full text of these articles, 73 articles met the inclusion criteria. Articles from the title and abstract screening stage were most often considered not relevant because the study was not relevant to both agricultural intensification and health (Figure 1). The inter-rater reliability for title/abstract screening and full-text screening was 0.95 and 0.91, respectively, indicating almost perfect agreement [18]. The majority of studies were conducted in Thailand (*n* = 32), followed by Vietnam (17), Philippines (7), Malaysia (6), and Cambodia (5). Few to no studies were conducted in the other Southeast Asian countries (6). Most of the articles (67%) were published within the last 5 years.

### Agricultural intensification associated health risks

Epidemiological approaches to directly assess health risks by measuring prevalence of disease or exposure were applied in 42 studies (Table 3). Of these, most studies assessed health risks from pesticide exposure [20–51], while other studies explored zoonotic diseases [52–55] (4), antimicrobial resistance (AMR) [56–58], or other health risks (heavy metals, parkinsonism, malaria) [59–61]. For instance, A cross-sectional study in Pangasinan, Philippines, found that a maximum of 20% of randomly selected eggplant samples tested positive for insecticide residues, some at levels exceeding the acceptable maximum residue limit set by the European Commission [22]. Farmers in the study (*n* = 58) reported experiencing itchiness of the skin (63.8%), redness of the eyes (29.3%), muscle pains (27.6%), and headaches (27.6%), as being related to their pesticide exposure. In a case-control study, factors influencing poisoning symptoms among 153 mixed insecticide-exposed vegetable farmers in one Cambodian village were identified [24]. This study found that mixing an average of four to six types of insecticides was associated with central nervous system symptoms (odds ratio [OR] = 4.6; *P* = .03), while organophosphate and carbamate use were associated with respiratory symptoms (OR = 3.2; *P* = .04). Sekiyama et al. [44] explored pesticide usage and its associated health symptoms among 73 farmers in West Java, Indonesia. Many of the subject farmers worked in a highly unsafe occupational environment and protective measures and safe handling were rarely observed. Correlation analysis revealed that wearing wet clothing (skin exposure to pesticide) and smoking during spraying were the significant determining factors for developing health symptoms (not specified). A cross-sectional study in Phu Tho, Vietnam, assessed potential exposure of local farmers and consumers to agrichemicals [32]. Recently used pesticides, such as fenobucarb, trichlorfon, cyfluthrin, and cypermethrin were detected in vegetable and fish samples. Thresholds for acceptable daily intake levels were frequently reached in the analyzed food products. A cross-sectional survey of 182 rice farmers and 122 controls in Thailand found that rice farmers had significantly higher prevalence of difficulty in breathing and chest pain compared to controls (OR = 2.8; *p* < 0.01, and OR = 2.5; *p* < 0.05, respectively). A health risk assessment related to dermal exposure of chlorpyrifos was conducted among 35 rice farmers in Nakhon Nayok Province, Thailand [25]. About 14% of rice farmers reported blurred vision and dizziness during pesticide application. The hazard quotient (HQ) at the mean and 95th percentile level was found to be greater than acceptable (HQ > 1); the authors concluded that rice-growing farmers in this area may be at risk for adverse health effects due to continuous dermal exposure to chlorpyrifos from their improper use of personal protective equipment.

**Table 3.** Summary of epidemiological studies that assessed health risks associated with agricultural intensification in Southeast Asia (*n* = 42).

Author and year	Study design	Target area	Exposure pathway	Specific health risk
Fiedler et al. [20]	CC study of 24 cases (Thai children living in farming community) and 29 controls	Bangkok, Thailand	Children living in rice farming community	No significant adverse neurobehavioral effects from organophosphates and chloropyrifos exposure
Chau et al. [21]	CS survey of 104 households living along canals	Can Tho and An Giang Provinces, Vietnam	Occupational (pesticide application), ingestion (drinking water sources)	Health risks from pesticide exposure (not specified)
Del Prado-Lu [22]	CS survey of eggplant farms (26 farms), with a total of 58 farmers and farm workers	Pangasinan Province, Philippines	Occupational (pesticide application), consumption (eggplants)	Pesticide-related symptoms (itchiness of the skin, redness of the eyes, muscle pains, headaches)
Rohitrattana et al. [23]	CC study of 24 cases (children living in rice farming communities) and 29 controls	Pathum Thani Province, Thailand	School-aged children living in rice and aquacultural farming regions	Health risks from organophosphate exposure (not specified)
Thetkathuek et al. [24]	CS survey of 153 mixed-insecticide exposed vegetable farmers	Kandal Province, Cambodia	Occupational (pesticide application)	Central nervous system, gastrointestinal, and respiratory symptoms from pesticide exposure
Lappharat et al. [25]	CS survey of 35 rice farmers, risk assessment, dermal sampling	Nakhon Nayok Province, Thailand	Occupational (pesticide application, dermal)	Pesticide-related symptoms (blurred vision, dizziness, headache, muscle weakness in arms and legs)
Ostrea et al. [26]	Cohort study of children enrolled at birth	Bulacan Province, Phillipines	Children of occupationally exposed parents (pesticide application)	Health risks from propoxur and pyrethroids exposure (not specified)
How et al. [27]	CC study of 95 cases (children who live near paddy farmland) and 85 controls	Selangor, Malaysia	Farm children who grow up near pesticide-treated farmland	Reduced blood cholinesterase level and risk for cancer from organophosphate exposure
Sapbamrer and Nata [28]	CC survey of 182 rice farmers (exposed subjects) and 122 non-farmers (controlled group)	Northern Thailand	Occupational (pesticide application)	Respiratory and muscle symptoms from pesticide exposure
Phung et al. [29]	CS survey of 18 rice farmers in Vu Le commune	Thai Binh Province, Vietnam	Occupational (chloropyrifos application)	Health risks associated with chloropyrifos exposure (not specified)
Lu [30]	CS survey of 400 vegetable farmers	Benguet, Philippine	Ingestion (vegetables), occupational (pesticide application)	Respiratory symptoms from pesticide exposure
Bhidayasiri et al. [31]	Retrospective analysis of Parkinson’s Disease Registry	Thailand	Urbanization and exposure to pesticides (not specified)	Parkinson’s disease from pesticide exposure
Hoai et al. [32]	CS survey of 54 farmers in Hoang Liet and Minh Dai communes	Hanoi and Phu Tho Province, Vietnam	Ingestion (fish, vegetables)	Health risks from pesticide exposure (not specified)
Wang et al. [33]	CS survey of 158 Cambodians	Kampong Cham, Kratie and Kandal Provinces, Cambodia	Ingestion (fish, vegetables)	Health risks from organochlorine pesticide exposure
Baharuddin et al. [34]	CS survey of 140 paddy farmers	Perak, Malaysia	Occupational (inhalation, dermal)	Health risks from pesticide exposure
Borkowski et al. [35]	CS survey of 9 mothers working in citrus orchids and 32 mothers (who do not work)	Chiang Mai Province, Thailand	Occupational (pesticide application)	Potential health risks from pesticide exposure to mothers and associated abnormal muscle problems of newborn infants
Prihartono et al. [36]	Hospital-based CC study in Thailand (541 cases of AA and 2261 controls)	Thailand	Occupational (pesticide application)	Risk for apastic anemia from pesticide exposure
Hanchenlaksh et al. [37]	CS survey of 16 randomly selected farmers’ families (8 vegetable and 8 fruit farmers)	Nakhonratchasima Province, Thailand	Occupational (pesticide application, farmers and household)	Health risks from pesticide exposure (not specified)
Hung et al. [38]	CS survey of 3814 individuals from 942 randomly selected households	Phu Tho Province, Vietnam	Occupational (pesticide application, farmers and household)	Pesticide poisoning
Hossain et al. [39]	CS survey of 152 male farmers	Sabah, Malaysia	Occupational (pesticide application)	Significant decline in semen quality and semen count from pesticide exposure
Jaipieam et al. [40]	CS survey of 33 vegetable farmers (case) and 17 farmers who do not work with pesticides (control)	Songkhla Province, Southern Thailand	Occupational (pesticide application, inhalation)	Health risks from chlorpyrifos and dicrotofos exposure (not specified)
Lu [41]	CS survey of 211 vegetable farmers and 37 farms	Benguet, Philippines	Occupational (pesticide application)	Pesticide-related symptoms
Kachaiyaphum et al. [42]	CS survey of 350 randomly selected chili-farm workers	Chaiyaphum Province, Thailand	Occupational (pesticide application)	Abnormal serum cholinesterase levels and pesticide related symptoms
Jintana et al. [43]	CC study of 90 cases (individuals occupationally exposed) and 30 controls	Rachaburi Province, Thailand	Occupational (pesticide application)	Inhibition of cholinesterases
Sekiyama et al. [44]	CS survey of 73 farmers	West Java, Indonesia	Occupational (pesticide application)	Occupational exposure to pesticides and self-reported symptoms
Lu [45]	CS survey of 114 cut-flower farmers	La trinidad, Philippines	(Occupational (pesticide application, ocular, dermal)	Abnormal cholinesterase level and other health risks associated with pesticide use
Tuc et al. [46]	CC study of 156 cases (rice farmers) and 314 controls	Thai Binh Province, Vietnam	Occupational (pesticide application)	Abnormal semen from pesticide exposure
Dasgupta et al. [47]	CS survey of 190 farmers in the Mekong Delta, Vietnam	Mekong Delta, Vietnam	Occupational (pesticide application)	Pesticide poisoning by organophosphate and carbamate exposure
Petchuay et al. [48]	CC study of 37 cases (farm children) and 17 cases	Songkhla Province, Southern Thailand	Children of occupationally exposed parents (pesticide application)	Health risk to farm children from organophosphate exposure (not specified)
Kunstadter et al. [49]	CS survey of 582 Highland Hmong Farmers	Chiang Mai, Thailand	Occupational (pesticide application)	Abnormal cholinesterase level from pesticide exposure
Jaipieam et al. [50]	CC study of 33 cases (vegetable growers) and 17 controls	Songkhla Province, Thailand	Ingestion (contaminated water), agricultural communities	Health risks from organophosphate exposure (not specified)
Riwthong et al. [51]	CS survey of 240 smallholder plant farmers	Chiang Mai, Chiang Rai and Nan Provinces, Thailand	Occupational (pesticide use)	Health risks from pesticide exposure (not specified)
Choe et al. [56]	CS survey of 12 pig farms	Seberang Perai, Malaysia	Pig farms	Antimicrobial resistant Salmonella spp. in finishing pigs
Tu et al. [57]	CS survey of 341 pig, chicken, and duck farms	Dong Thap Province, Vietnam	Duck farms, pig farms, farms with frequent rodent sightings	Antimicrobial resistant non-typhoidal Salmonella serovars
Patchanee et al. [58]	CS survey of 104 pig farms	Chiang Mai and Lamphun Provinces, Thailand	Pig farms	Antimicrobial resistant MRSA
Pattanasin et al. [59]	CS survey of 394 randomly selected rubber tapper households.	Prachuab Khiri Khan Province, Thailand	Occupational exposure of rubber tapper farmers and their families	Malaria
Tarafder et al. [52]	CS survey of 25 rain-fed and 25 irrigated villages endemic for *Schistosoma japonicum*	Samar Province, Philippines	Occupational (rice farming)	Possible association with *S. japonicum* infection
Tangkanakul et al. [53]	CC study of 59 cases (leptospirosis patients) and 118 controls	Nakornratchasrima Province, Thailand	Occupational (applying fertilizer in wet fields, walking through water)	Leptospirosis
Watthanakulpanich et al. [54]	CC study of 58 cases (those infected with Trichostrongyliasis) and 58 controls	Savannakhet Province, Laos	Ingestion (regular consumption of fresh vegetables), lack of hand washing, and close contact with cattle	Trichostrongyliasis
Bless et al. [55]	CS survey of 257 schoolchildren	Kandal Province, Cambodia	• Ingestion (raw aquatic vegetables) • Zoonotic	• Large trematode eggs in the stool • Possible *Fasciola spp.* transmission from cattle to human
Munisamy et al. [60]	CS survey of 87 vegetable farmers	Cameron Highlands, Malaysia	Ingestion (vegetables)	There are unlikely potential adverse health impacts arising from Cadmium through vegetables consumption
Norkaew et al. [61]	CS survey of 90 elderly people living in an agricultural community	Ubon Ratchathani Province, Thailand	Elderly people living in agricultural community	Parkinsonism

Abbreviations: CC, case-control; CS, cross-sectional.

The prevalence and AMR of non-typhoidal Salmonella and associated risk factors was investigated among 341 pig, chicken, and duck farms in Dong Thap Province, Vietnam [57]. The farm-level adjusted non-typhoidal Samonella prevalence was 64.7, 94.3, and 91.3% for chicken, duck, and pig farms, respectively. Isolates had a high prevalence of resistance (77.6%) against tetracycline, and moderate resistance (20–30%) against chloramphenicol, sulfamethoxazole-trimethoprim, ampicillin, and nalidixic acid. Methicillin-resistant *Staphylococcus aureus* (MRSA) was investigated in 104 pig farms in Northern Thailand [58]. Herd prevalence of MRSA was 9.61% and antimicrobial sensitivity tests found 100% of the MRSA isolates resistant to clindamycin, oxytetracycline, and tetracycline. The authors remarked that this is the first evidence of a livestock-associated MRSA interrelationship among pigs, workers, and the farm environment in Thailand. A case-control study (59 cases) was conducted in Nakornratchasrima, Province, to determine risk factors for leptospirosis [53]. Factors associated with leptospirosis infection include: walking through water (OR = 4.9, 95%), and applying fertilizer in wet fields for more than 6 h a day (OR = 3.4).

### Contaminants and exposure routes

An additional 31 studies indirectly assessed health risks using methods such as health risk assessments, and testing levels of environmental or food contamination and comparing to established guidelines (e.g. WHO, FAO, European Union guidelines) (See Appendix: Supplementary Table S1). However, this information may be limited due to assumptions in risk estimates, and varying consumer exposure scenarios. Contaminant uptake, especially pesticides, in the food chain and drinking water supply was a key focus of studies captured in this review. Heavy metal (5) and microbial (4) contamination of food and water supply were also explored, along with the emergence of AMR pathogens (5), insecticide resistant vectors (3), and zoonotic diseases (2). For example, In Nakhon Pathom Province, Thailand, pesticide residues were found in Chinese kale samples (*n* = 117) in markets [62]. Of the 28 pesticides investigated, 12 pesticides were detected in 85% of the samples. Further, in 34 samples tested, either carbofuran, chlorpyrifos, chlorothalonil, cypermethrin, dimethoate, metalaxyl, or profenofos was detected and exceeded their maximum residue levels. The levels of pollution by organic pollutants, metals, and microbial indicators were determined in the Mekong Delta area, Vietnam [63]. Ammonium, arsenic, barium, chromium, mercury, manganese, aluminum, *Escherichia coli* and total coliforms in canals exceed thresholds set by Vietnamese quality guidelines for drinking water and domestic purposes. *Salmonella spp.* isolated in pig production lines both pig farms and from slaughterhouses were characterized in Northern Thailand [64]. A total of 86 strains of Salmonella comprising five majority serotypes were identified, and antibiotic resistance to tetracycline was found to be the most prevalent (82.56%) followed by ampicillin (81.40%) and streptomycin (63.95%). Insecticide resistance was detected among *Aedes Aegypti* adults in Singapore [65] and *Culex Vishnui* in Malaysia [66], revealing the effects of agricultural insecticide pressures on vectors.

Not all studies, however, identified risks associated with agricultural intensification [62,67–69]. For example, in Hanoi, Vietnam, the concentrations of heavy metals in soil and water spinach cultivated with wastewater was explored [67]. The estimated average daily intake of As, Cd, Cu, Fe, Pb, and Zn for adult Vietnamese consumers was below the maximum tolerable intake proposed by FAO/WHO for each element. The authors conclude that the occurrence of the investigated elements in water spinach will pose low health risk for the consumers. Moreover, in Indonesia, a total of 23 organochlorine pesticides residues were determined in five groups of foodstuffs collected from traditional markets [68]. Very low concentrations of organochlorine pesticide residues were detected in foodstuffs (far below the maximum residue limits as established by FAO/WHO), and the estimated daily intake were far below the acceptable daily intake as established by FAO/WHO. The authors suggest that consumption of foodstuffs from Indonesia were of little risk to human health in term of organochlorine pesticides.

## Discussion

Agricultural intensification is expected to continue to expand in order to meet the global demand for food security. Understanding the major health risks and exposure pathways is crucial in making sustainable agricultural management decisions. Our review summarized and synthesized the results from 73 studies investigating the human health risks associated with agricultural intensification in Southeast Asia. Overall, studies on human health risks appear to be limited and almost exclusively focused on pesticides. Indeed, pesticides are widely used in agricultural sectors in many countries in Southeast Asia to maintain high agricultural yields. Exposure to pesticides among farmers, children living in agricultural areas, and consumers, was a large concern identified in this review. Moreover, chemicals have been extensively used to eradicate vector borne diseases, leading to the emergence of insecticide resistant vectors [65,66,70]. Few studies explored risks from zoonotic disease emergence [52–55], contributing to the growing evidence of zoonosis emergence linked to agricultural intensification [71]. One study explored the linkage between malaria and rubber tapper farmers [59]. Indeed, Southeast Asian countries are the world’s largest producers of natural rubber products, and agricultural workers on rubber plantations are particularly at risk for malaria and other vector borne diseases due to land transformations, climatic conditions, and vector population dynamics [72].

A common exposure pathway analyzed was the consumption of crops contaminated with pesticides, heavy metals, or microbial contaminants. Studies mainly compared contaminant levels to guidelines, and few studies found that contaminant levels did not exceed guidelines. Yet, contaminants are also present in the environment (e.g. drinking water), and so studies must also consider other environmental health risks when comparing contaminant levels to guidelines. For example, Marcussen et al. [67] found that the estimated average daily intake of heavy metals for Vietnamese consumers of water spinach was below dietary thresholds (based on estimated daily intake proposed by FAO/WHO). However, Wilbers et al. [63] found that heavy metal concentrations in canals exceeded the thresholds set by Vietnamese quality guidelines for drinking and domestic purposes. The use of human waste, animal waste, and wastewater is an important occupational health risk and contributor to contamination of crops [9]. As such, the importance of compounding exposures such as water, soil, occupational exposures (e.g. use of pesticides, waste, and waste by-products), and community members living in agricultural areas, needs to be considered in studies in order to accurately determine health risks.

More than half of the study designs were cross-sectional (29 out of 42 epidemiology studies), in which exposure and outcome were assessed at the same point of time. This study design posed limitations in drawing causal inferences from the results as these studies could not determine whether exposure occurred before, during, or after the onset of poor health outcome. Only one study used a cohort study design to describe the trend in long-term pesticide exposure [26]. As such, the chronic health outcomes among consumers of contaminated sources and farmers are more difficult to attribute to agricultural intensification, and more long-term studies are needed to increase understanding of these health risks. Furthermore, a better understanding of the benefits of agricultural intensification, along with mitigation strategies, will help inform agricultural management decisions. For example, a recent study assessed for potential health impacts of waste recovery and reuse business models in Hanoi, Vietnam [73]. Future research should explore benefits provided by agricultural practices together with the risks, along with mitigation strategies in order to better inform agricultural decisions that protect public health.

Research to characterize knowledge and attitudes relating to agricultural practices and health risks will provide opportunities to identify useful public health interventions to mitigate health risks. Several recent qualitative studies (not summarized in this review) have explored health risk perceptions of pesticide use and associated influence on behavior [74–77]. For example, There was a high level of awareness on the negative impacts of commercial pesticides and fertilizers on soil, water and human health but awareness did not influence the way farmers utilize pesticides and dispose of empty bottles/containers of pesticides after use [76]. In another study, the importance of using personal protective equipment is known and well understood by the farmers. However, in practice, only 3.8% of farmers wore protective glasses and 1.9% were using boots, indicating a gap between knowledge and practice. Understanding risk perceptions associated with agricultural intensification is an important component of the health risk assessment, and in designing interventions to promote safe agricultural practices.

We found that little research has been done to understand the complex risk factors associated with agricultural intensification, such as evaluating multiple contaminant groups, and multiple exposure pathways. Similarly, a recent systematic review assessed for health risks of agricultural intensification in the Mekong Delta [78] found that little research has been done on this issue and no efforts have been made to consolidate the health risks from the simultaneous exposures to a range of hazardous chemicals used. The authors concluded that while some of the studies identified environmental contamination bearing considerate health risks, efforts are needed to consolidate the health risks from regional intensification measures. A meta-analysis of adverse health effects due to agricultural intensification may improve the strength of evidence.

## Conclusion

This review found evidence of health risks from occupational exposure to pesticides and consumption of pesticide-contaminated food and drinking water. Several studies also determined heavy metal and microbiological contamination of food and drinking water from agricultural inputs. Furthermore, research on AMR pathogens, insecticide-resistant vectors, and zoonotic diseases, have emerged over recent years. From this review, it appears that agricultural intensification is having a broad impact on communities, ranging from agricultural workers, to those living in an agriculture area and consumers; however, many studies focused on exposure pathways without specifying the health risk, and lacked a long-term study design. We argue that intensification is still largely concerned with how to enhance agricultural productivity while reducing its environmental impacts, but not enough focus on the implications on health and livelihoods. More research to determine long-term health risks associated with agricultural intensification; explore multiple exposure routes and contaminants; and strategies to mitigate health risks; are warranted in order to inform agricultural management decisions that improve food security while protecting the environment and public health.

## Funding

The International Development Research Centre (IDRC), Canada, funded this research through the [grant number 106556] for the project “Ecohealth Field Building Leadership Initiative in Southeast Asia (FBLI)”. HNV was partly funded by the CGIAR research program on Agriculture for Nutrition and Health (A4NH).

## Disclosure statement

No potential conflict of interest was reported by the authors.

## Supplemental data

Supplemental data for this article can be accessed at https://doi.org/10.1080/10773525.2018.1450923.

## Supplementary Material

YJOH_A_1450923_Supplementary_material.docx
