# Supplementary material for: Emerging health risks from agricultural intensification in Southeast Asia: a systematic review
Source: Int J Occup Environ Health. 2018 Mar 21;23(3):250–60. doi: 10.1080/10773525.2018.1450923 (PMC6060873; doi:10.1080/10773525.2018.1450923)
Supplement: YJOH_A_1450923_Supplementary_material.docx [file YJOH_A_1450923_SM1827.docx]

**Appendix: Supplementary table**

Emerging health risks from agricultural intensification in Southeast Asia: a systematic review

Steven Lam, Giang Pham, Hung Nguyen-Viet

**Table S1.** Description of studies using indirect methods to assess health risks of agricultural intensification in Southeast Asia (n=31).

| Author and year | Study design | Target area | Exposure route | Health risk |
| --- | --- | --- | --- | --- |
| Garcia et al. 2015 ^1^ | Water, soil, and vegetable sampling | Manila, Philippines | Ingestion of vegetables | Biological (*Escherichia coli*, somatic coliphages, *Salmonella spp.*) |
| Keawvichit et al. 2001 ^2^ | Water and soil sampling | Chiang Mai, Thailand | Ingestion of vegetables | Parasitic and biological (unspecified) |
| Diallo et al. 2008 ^3^ | Quantitative microbial risk assessment | Pathum Thani Province, Thailand | Use of canal water for recreational purposes or occupational (use of unrestricted and restricted irrigation) | Parasitic (giardiasis) and biological (unspecified) |
| Hanh et al. 2010 ^4^ | Water sampling | 5 provinces in Vietnam | Ingestion of drinking water | Biological (total coliform, faecal coliform) |
| Wanwimolruk et al. 2015 ^5^ | Chinese kale sampling | Nakhon Pathom Province, Thailand | Ingestion of vegetables | Pesticides |
| Siriwong et al. 2009 ^6^ | Dermal sampling of fisherman, health risk assessment | Rangsit, Pathum Thani Province, Thailand | Occupational (pesticide application, dermal) | Pesticides and cancer |
| Siriwong et al. 2008 ^7^ | Vegetable and fish sampling, health risk assessment | Pathum Thani Province, Thailand | Ingestion of fish and vegetables | Pesticides |
| Wanwimolruk et al. 2015 ^8^ | Fruit sampling | 8 provinces in Thailand | Ingestion of fruits | Pesticides |
| Phung 2013 ^9^ | Health risk assessment, urine sampling | Vietnam | Occupational (pesticide application) | Pesticides |
| Shoiful et al. 2013 ^10^ | Vegetable sampling | Jakarta, Bogor, and Yogyakarta cities in Indonesia | Ingestion of vegetables | Pesticides |
| Phung et al. 2012 ^11^ | Urine sampling | Thai Binh Province, Vietnam | Occupational (pesticide application) | Pesticides |
| Panuwet et al. 2009 ^12^ | Urine sampling | Chiang Mai Province, Thailand | Ingestion (not specified) | Pesticides |
| Jirachaiyabhas et al. 2004 ^13^ | Exposure assessment, air sampling | Tambon Bang Rieng, Thailand | Occupational (pesticide application, inhalation) | Pesticides |
| Minh et al. 2004 ^14^ | Human breast milk sampling | Hanoi and Ho Chi Minh city, Vietnam | Ingestion of breastmilk | Pesticides |
| Wilbers et al. 2014 ^15^ | Mekong river surface water sampling | Mekong Delta, Vietnam | Ingestion of drinking water | Heavy metals |
| Seyfferth et al. 2014 ^16^ | Rice paddy fields sampling | Five provinces in Cambodia | Ingestion of rice | Heavy metals |
| Marcussen et al. 2008 ^17^ | Water spinach and soil sampling | Hanoi, Vietnam | Ingestion of vegetables | Heavy metals |
| Kurosawa et al. 2006 ^18^ | Ground water and surface water sampling | Hanoi, Vietnam | Ingestion of drinking water | Heavy metals |
| Kurosawa et al. 2004 ^19^ | Ground water and surface water sampling | Red River Delta, Vietnam | Ingestion of drinking water | Heavy metals |
| Paul et al. 2010 ^20^ | Analysis of outbreak data | Thailand | Occupational (rice-cropping) | Highly pathogenic avian influenza (HPAI) H5N1 virus |
| Kandun 2010 ^21^ | Outbreak investigation | Indonesia | Occupational (using poultry faeces as garden fertilizer) | HPAI H5N1 virus |
| Koou et al. 2014 ^22^ | *Aedes aegypti* mosquitoes sampling, insecticide susceptibility testing | Singapore | N/A | Insecticide resistant vectors |
| Leong et al. 2014 ^23^ | *Culex vishnui* mosquitoes sampling, insecticide susceptibility testing | Selangor, Malaysia | N/A | Insecticide resistant vectors |
| Overgaard et al. 2005 ^24^ | Anopheline mosquitoes sampling, insecticide susceptibility testing | Chiang Mai, Thailand | N/A | Insecticide resistant vectors |
| Wang et al. 2014 ^25^ | Faecal sampling (pigs, humans) | Cambodia | N/A | Zoonotic transmission of *Blastocystis spp.* from close contact with pigs |
| Olaitan et al. 2015 ^26^ | Faecal sampling (humans, pigs, goats), antimicrobial sensitivity testing | Laos | N/A | Zoonotic transmission of a colistin-resistant *E. coli* from close contact with pigs |
| Tadee et al. 2015 ^27^ | Sampling at pig farms and slaughterhouses, antimicrobial sensitivity testing | Chiang Mai and Lamphun provinces in northern Thailand | N/A | AMR (*Salmonella spp.*) in pig farms |
| Dang et al. 2011 ^28^ | Water-sediment and pig manure sampling, antimicrobial sensitivity testing | Hanoi, Vietnam | N/A | AMR (*E. coli* and *Enterococcus spp.)* in integrated animal husbandry-fish farms |
| Chotinun et al. 2014 ^29^ | Sampling around slaughterhouses, antimicrobial sensitivity testing | Chiang Mai, Thailand | N/A | AMR (*Salmonella spp.*) in poultry farms |
| Thai et al. 2012 ^30^ | Pork and chicken meat sampling, antimicrobial sensitivity testing | Bac Ninh Province, Ha Tay Province, and Hanoi, Vietnam | N/A | AMR (*Salmonella spp.*) in pig farms |
| Larsen et al. 2012 ^31^ | Sampling nasal swab of pigs and antimicrobial sensitivity testing | Chiang Mai Province, Thailand | N/A | AMR (Methicillin-resistant *Staphylococcus aureus*) in pig farms |

**References**

1. Garcia BCB, Dimasupil MAZ, Vital PG, Widmer KW, Rivera WL. Fecal contamination in irrigation water and microbial quality of vegetable primary production in urban farms of Metro Manila, Philippines. *J Environ Sci Health B*. 2015;50(10):734-743.

2. Keawvichit R, Wongworapat K, Putsyainant P, Silprasert A, Karnchanawong S. Parasitic and bacterial contamination in collards using effluent from treated domestic wastewater in Chiang Mai, Thailand. *Southeast Asian J Trop Med Public Health*. 2001;32(SUPPL. 2):240-244.

3. Diallo MBC, Anceno AJ, Tawatsupa B, Houpt ER, Wangsuphachart V, Shipin O V. Infection risk assessment of diarrhea-related pathogens in a tropical canal network. *Sci Total Environ*. 2008;407(1):223-232.

4. Hanh PTM, Sthiannopkao S, Kim KW, Ba DT, Hung NQ. Anthropogenic influence on surface water quality of the Nhue and Day sub-river systems in Vietnam. *Environ Geochem Health*. 2010;32(3):227-236.

5. Wanwimolruk S, Kanchanamayoon O, Phopin K, Prachayasittikul V. Food safety in Thailand 2: Pesticide residues found in Chinese kale (Brassica oleracea), a commonly consumed vegetable in Asian countries. *Sci Total Environ*. 2015;532:447-455.

6. Siriwong W, Thirakhupt K, Sitticharoenchai D, et al. Risk Assessment for Dermal Exposure of Organochlorine Pesticides for Local Fishermen in the Rangsit Agricultural Area, Central Thailand. *Hum Ecol RISK Assess*. 2009;15(3):636-646.

7. Siriwong W, Thirakhupt K, Sitticharoenchai D, et al. A Preliminary Human Health Risk Assessment of Organochlorine Pesticide Residues Associated with Aquatic Organisms from the Rangsit Agricultural Area, Central Thailand. *Hum Ecol RISK Assess*. 2008;14(5):1086-1097.

8. Wanwimolruk S, Kanchanamayoon O, Boonpangrak S, Prachayasittikul V. Food Safety in Thailand 1: It is Safe to Eat Watermelon and Durian in Thailand. *Environ Health Prev Med*. 2015:204-215.

9. Phung DT, Connell D, Yu Q, Chu C. Health risk characterization of chlorpyrifos using epidemiological dose-response data and probabilistic techniques: A case study with rice farmers in Vietnam. *Risk Anal*. 2013;33(9):1596-1607.

10. Shoiful A, Fujita H, Watanabe I, Honda K. Concentrations of organochlorine pesticides (OCPs) residues in foodstuffs collected from traditional markets in Indonesia. *Chemosphere*. 2013;90(5):1742-1750.

11. Phung DT, Connell D, Miller G, et al. Biological monitoring of chlorpyrifos exposure to rice farmers in Vietnam. *Chemosphere*. 2012;87(4):294-300.

12. Panuwet P, Prapamontol T, Chantara S, Barr DB. Urinary pesticide metabolites in school students from northern Thailand. *Int J Hyg Environ Health*. 2009;212(3):288-297.

13. Jirachaiyabhas V, Visuthismajarn P, Hore P, Robson MG. Organophosphate pesticide exposures of traditional and integrated pest management farmers from working air conditions: A case study in Thailand. *Int J Occup Environ Health*. 2004;10(3):289-295.

14. Minh NH, Someya M, Minh TB, et al. Persistent organochlorine residues in human breast milk from Hanoi and Ho chi minh city, Vietnam: Contamination, accumulation kinetics and risk assessment for infants. *Environ Pollut*. 2004;129(3):431-441.

15. Wilbers G-J, Becker M, Nga LT, Sebesvari Z, Renaud FG. Spatial and temporal variability of surface water pollution in the Mekong Delta, Vietnam. *Sci Total Environ*. 2014;485-486:653-665.

16. Seyfferth AL, McCurdy S, Schaefer M V., Fendorf S. Arsenic concentrations in paddy soil and rice and health implications for major rice-growing regions of cambodia. *Environ Sci Technol*. 2014;48(9):4699-4706.

17. Marcussen H, Joergensen K, Holm PE, Brocca D, Simmons RW, Dalsgaard A. Element contents and food safety of water spinach (Ipomoea aquatica Forssk.) cultivated with wastewater in Hanoi, Vietnam. *Environ Monit Assess*. 2008;139(1-3):77-91.

18. Kurosawa K, Hai Nguyen D, Thanh Huu N, Le Ha Thi T, Egashira K. Quality of groundwater and surface water in terms of inorganic nitrogen concentration in an urbanized agricultural area around Ha Noi, Viet Nam. *Japanese J Trop Agric*. 2006;50(1):15-22.

19. Kurosawa K, Han DN, Thanh NH, Tra HTL, Canh NT, Egashira K. Monitoring of inorganic nitrogen levels in the surface and ground water of the Red River Delta, northern Vietnam. *Commun Soil Sci Plant Anal*. 2004;35(11-12):1645-1662.

20. Paul M, Tavornpanich S, Abrial D, et al. Anthropogenic factors and the risk of highly pathogenic avian influenza H5N1: prospects from a spatial-based model. *Vet Res*. 2010;41(3):28.

21. Kandun IN, Samaan G, Harun S, et al. Chicken faeces garden fertilizer: Possible source of human avian influenza H5N1 infection. *Zoonoses Public Health*. 2010;57(4):285-290.

22. Koou S-Y, Chong C-S, Vythilingam I, Lee C-Y, Ng L-C. Insecticide resistance and its underlying mechanisms in field populations of Aedes aegypti adults (Diptera: Culicidae) in Singapore. *Parasit Vectors*. 2014;7:471.

23. Leong CS, Chen CD, Low VL, Karen-Chia HM, Azidah AA, Sofian-Azirun M. Multiple resistance of Culex vishnui Theobald against four major classes of insecticides in an agricultural area in Sekinchan, Selangor, Malaysia. *Trop Biomed*. 2014;31(2):241-260.

24. Overgaard HJ, Sandve SR, Suwonkerd W. Evidence of anopheline mosquito resistance to agrochemicals in northern Thailand. *Southeast Asian J Trop Med public Heal*. 2005;36 Suppl 4:152-157.

25. Wang W, Owen H, Traub RJ, Cuttell L, Inpankaew T, Bielefeldt-Ohmann H. Molecular epidemiology of Blastocystis in pigs and their in-contact humans in Southeast Queensland, Australia, and Cambodia. *Vet Parasitol*. 2014;203(3-4):264-269.

26. Olaitan AO, Thongmalayvong B, Akkhavong K, et al. Clonal transmission of a colistin-resistant Escherichia coli from a domesticated pig to a human in Laos. *J Antimicrob Chemother*. 2015;70(12):1-2.

27. Tadee P, Boonkhot P, Pornruangwong S, Patchanee P. Comparative phenotypic and genotypic characterization of Salmonella spp. in pig farms and slaughterhouses in two provinces in northern Thailand. *PLoS One*. 2015;10(2):e0116581.

28. Dang STT, Petersen A, Van Truong D, Chu HTT, Dalsgaard A. Impact of medicated feed on the development of antimicrobial resistance in bacteria at integrated pig-fish farms in Vietnam. *Appl Environ Microbiol*. 2011;77(13):4494-4498.

29. Chotinun S, Rojanasthien S, Unger F, Tadee P, Patchanee P. PREVALENCE AND ANTIMICROBIAL RESISTANCE OF SALMONELLA ISOLATED FROM CARCASSES, PROCESSING FACILITIES AND THE ENVIRONMENT SURROUNDING SMALL SCALE POULTRY SLAUGHTERHOUSES IN THAILAND. *Southeast Asian J Trop Med Public Health*. 2014;45(6):1392-1400.

30. Thai TH, Hirai T, Lan NT, Yamaguchi R. Antibiotic resistance profiles of Salmonella serovars isolated from retail pork and chicken meat in North Vietnam. *Int J Food Microbiol*. 2012;156(2):147-151.

31. Larsen J, Imanishi M, Hinjoy S, et al. Methicillin-resistant staphylococcus aureus st9 in pigs in thailand. *PLoS One*. 2012;7(2):e31245.
